# Pregnancy and Delivery during Complete Prolapsus of the Uterus

**Published:** 1847-02

**Authors:** 


*Pregnancy and Delivery during complete Prolapsus of the Uterus.*—In the "Bull. delle Sc. Med. di Bologna," is recorded the case of a female, who first suffered from prolapsus of the uterus in her fifteenth year. In her 22d year she became pregnant, and her pregnancy proceeded to the seventh month, without any serious inconvenience. In the seventh month, the uterus began to sink, and, in the commencement of the eighth month, it projected as much as six fingers' breadth beyond the vulva. The author was not called until the patient had been four days in labor. When he arrived he found the woman much exhausted, and the organ of a dark brown color, hanging as low down as the middle third of the thigh. Labor proceeded in the usual way; the child was extracted with forceps; and, on the twentieth day, the woman was able to leave her bed. —*Ancell's Report, in Ranking's Abstract*, vol iii.

